# The Book World of Medicine and Science

**Published:** 1895-05-18

**Authors:** 


### The Book World of Medicine and Science.

Elements of Surgical Pathology. By Augustus J. Pepper, M.S., M.B Lond., F.R.C.S.Eng., Surgeon to St. Mary's Hospital. Illustrated with 99 Engravings. Fourth Edition. (London: Cassell and Co., 1894. Price 8s. 6d.)

This edition is rewritten and enlarged, several diseases being included which found no place in the last edition. Pepper's "Pathology" is so well known, and is so popular a book that there is no need to refer in detail to its special features. It deals with a very vast subject, and one that is constantly growing, and as the space at the author's command is limited, it follows that many of the descriptions are very compressed and even scanty. This sometimes leads to a certain lack of clearness, and we can imagine students who are not familiar with the subject being rather puzzled by some portions of the chapter on caries, for instance. The part taken by micro-organisms in many pathological processes is so important that we turned with special interest to see how fully Mr. Pepper dealt with this part of his subject. We must confess to a certain degree of disappointment, and we hope that in the next edition considerably more attention may be paid to surgical bacteriology. Appendicitis is one of the additions specially referred to in the preface to this volume, and here again we think Mr. Pepper has suffered the limitation of space to mar his work. We find the barest outline of the pathology of this very important disease, and yet a tolerably intimate acquaintance with it is necessary for the prompt recognition and wise treatment of the affection. It appears to us that too much has been attempted in the space allotted. The book is too complete in the number of affections dealt with, and this has been secured at the expense of a sufficient treatment of the great pathological processes. It is impossible not to be struck, however, with the great accuracy and wide and practical knowledge of his subject Mr. Pepper everywhere displays.

Drainage Work and Sanitary Fittings. By W. H. Maxwell. 74 pp. Price 1s. (St. Bride's Press. 24, Bride Lane, E.C.)

This book explains concisely and with tolerable completeness the methods of dealing with sewage, constructing sewers and calculating their capacities, and of laying, examining, and testing house-drains, but occasionally touches without explaining subjects requiring elucidation. Thus the author condemns cesspools, and advocates instead a "solids intercepting chamber" (vaguely described as "small") and the "treatment of the effluent by the polarite process" without explaining the construction of the one or the nature of the other. Again, "grease-traps" are condemned, but no treatment suggested for what is, admittedly, a difficult problem. The diagram on page 26 is needlessly obscure; and the ventilation of drains in the direction of their current may mislead, without any caution as to the relative lengths of downcasts and up-casts necessary to secure efficiency. The chapter on testing drains and sanitary fittings is excellent.

